# Neural Networks Mediating Perceptual Learning in Congenital Blindness

**DOI:** 10.1038/s41598-019-57217-w

**Published:** 2020-01-16

**Authors:** Daniel-Robert Chebat, Fabien C. Schneider, Maurice Ptito

**Affiliations:** 1 Visual and Cognitive Neuroscience Laboratory (VCN Lab), Department of Psychology, Faculty of Social Sciences and Humanities, Ariel University, Ariel, Israel; 2 Navigation and Accessibility Research Center of Ariel University (NARCA), Ariel, Israel; 3 University of Lyon, Saint-Etienne, F-42023 France; 4 Neuroradiology Unit, University Hospital of Saint-Etienne, Saint-Etienne, France; 5 BRAINlab, Department of Neuroscience and Pharmacology, University of Copenhagen, Copenhagen, Denmark; 6 Chaire de recherche Harland Sanders en Sciences de la Vision, École d’Optométrie, Université de Montréal, Montréal, Canada

**Keywords:** Human behaviour, Cognitive neuroscience, Human behaviour, Human behaviour, Cognitive neuroscience

## Abstract

Despite the fact that complete visual deprivation leads to volumetric reductions in brain structures associated with spatial learning, blind individuals are still able to navigate. The neural structures involved in this function are not fully understood. Our study aims to correlate the performance of congenitally blind individuals (CB) and blindfolded sighted controls (SC) in a life-size obstacle-course using a visual-to-tactile sensory substitution device, with the size of brain structures (voxel based morphometry-VBM-) measured through structural magnetic resonance Imaging (MRI). VBM was used to extract grey matter volumes within several a-priori defined brain regions in all participants. Principal component analysis was utilized to group brain regions in factors and orthogonalize brain volumes. Regression analyses were then performed to link learning abilities to these factors. We found that (1) both CB and SC were able to learn to detect and avoid obstacles; (2) their learning rates for obstacle detection and avoidance correlated significantly with the volume of brain structures known to be involved in spatial skills. There is a similar relation between regions of the dorsal stream network and avoidance for both SC and CB whereas for detection, SC rely more on medial temporal lobe structures and CB on sensorimotor areas.

## Introduction

Vision is undoubtedly a great facilitator of navigational tasks^1^ (for review see^2^). While approaching an obstacle, visual cues regulate foot placements by providing constant spatial updates of the distance to the obstacle^3^ in order to adapt locomotor behavior^4^. In sighted people this behavior is mediated by a complex network of interacting brain regions that integrates visual information and translates visual cues into appropriate behavior^5^. The hippocampal formation and the posterior parietal cortex are traditionally thought to play a pivotal role in navigation^6^. These two brain regions are involved in the processing of higher order spatial-cognitive information^7^, and in the registering of spatial information which is crucial for navigation^8^. They are largely affected by the absence of vision from birth and might therefore interfere with the blind navigational skills^9–11^. Indeed, spatial abilities in auditory and tactile spatial tasks are slightly compromised by early blindness^12,13^. A possible explanation for these compromised spatial abilities is that in the absence of vision, the neuronal networks responsible for spatial tasks do not develop in the same way as in the sighted^14^. All the components of the visual system in congenitally blind (CB) are volumetrically reduced^15–17^. In addition, a cascade of other non-visual brain structures undergo anatomical^18^, morphological^19^, and morphometric^20–23^ alterations, as well as modifications in functional connectivity^24^. There is no doubt that the brain of born blind individuals undergoes substantial reorganization compared to the sighted and is still able to carry out a number of behavioral tasks including navigation see^25,26^ for recent reviews with the use of sensory substitution devices (SSDs).

SSDs can potentially help make navigation easier for people who are visually impaired or CB by providing visual information via the auditory or tactile channels^27–30^. Indeed, CB have preserved navigational skills in an obstacle course^27^, that are associated with the activation of the hippocampal/parahippocampal area using fMRI^31^, and are also capable of completing and integrating paths (for review see^32^). Furthermore, it has been demonstrated that CB are able to generate cognitive representations of space stemming from the remaining intact senses^28–31^ and they preserve the ability to recognize a travelled route and represent spatial information mentally^31^. They even transfer spatial knowledge from virtual reality to the real world and vice versa^28,29^. CBs’ preserved navigational skills correlate with a larger anterior hippocampus^10^ that is accompanied by a volume reduction of its posterior portion^9^. These findings were explained by the possibility that the blind may rely more heavily on structures other than the hippocampus (such as the posterior parietal cortex) for navigation (^31^^,^^25^). The taxing demands of learning to detect and avoid obstacles without vision may drive hippocampal plasticity and volumetric changes in CB^9,11,15,32^. The precise hippocampal cellular layers concerned by this plasticity remain however unknown.

In this study, we provide comparative behavioral results on the learning rates of CB vs sighted control (SC) equipped with a Visual-to-tactile sensory substitution system (the tongue Display Unit or TDU), in an obstacle course. Using principal component analysis (PCA), we explore the relationship between learning performances of CB and SC individuals and some specific brain areas known to be involved in navigation.

## Results

All participants could ‘see’ objects in front of them with the device. Participants performed a ‘visual’-tactile acuity test and were ascribed a score^33^. We found that there is a significant correlation between the performance of our CB participants and their ‘visual’-tactile acuity score in the training part of the experiment for obstacle detection (r(32) = 0.286; p < 0.05), and for avoidance (r(32) = 0.314; p < 0.05). There was no correlation however between the ‘visual’-tactile acuity score in the test phase of the experiment for detection (r(32) = 0.031; p > 0.05), or avoidance (r(32) = 0.216; p > 0.05).

### Learning rate

**Figure 1:**
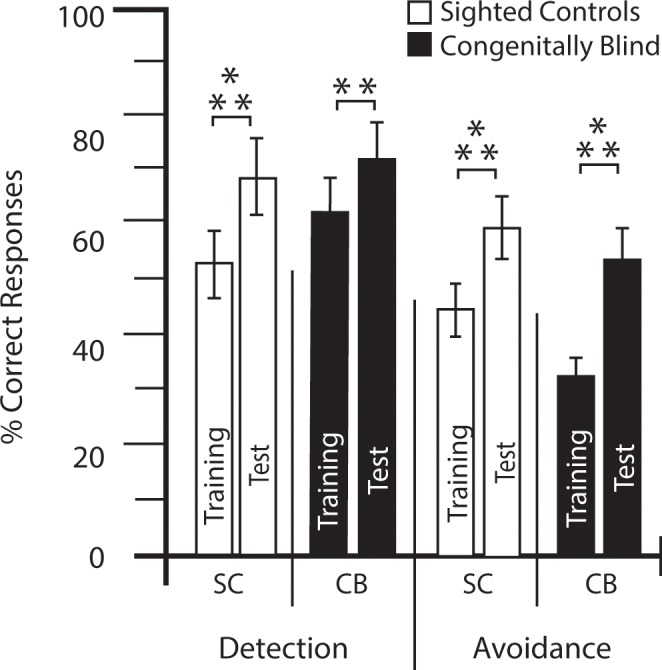
Performance. Bar graph comparing performances (percent correct response) for detection and avoidance in CB and SC participants for the training and test parts of the experiment. **P ≤ 0.01; ***P ≤ 0.001.

All participants were able to describe the distance (near, far) and type of obstacles in the training phase of the experiment. They could also accurately point to and avoid the obstacles. Both SC and CB groups significantly improved in terms of detection and avoidance between the training and test phases of the experiment (Fig. 1). There were significant differences in the scores for detection in the training phase (SC: M = 53.48, SE = 4.15; CB: M = 62.18, SE = 5.72) and in the test phase (SC: M = 68.57, SE = 5.23; CB: M = 71.64, SE = ; 4.56) of the experiment (SC: t(20) = 7.067, p = 0.000; CB: t(11) = 3.506, p = 0.002). There were also significant differences in the scores for avoidance in the training phase (SC: M = 45.38, SE = 3.74; CB: M = 33, SE = 3.83) and in the test phase (SC: M = 59.86, SE = 3.99; CB: M = 54.09, SE = 6.67) of the experiment (SC: t(20) = 7.406, p = 0.000; CB: t(11) = 3.94, p = 0.001) (see Fig. 1).

Learning rate was calculated individually for each subject as the difference between the training and testing phases of the experiment on the scores for detection and avoidance. Only one SC participant did not improve between the training phase and testing phase of the experiment (detection: SC M = 15.10 SE = 2.12), and all SC participants improved in terms of avoidance (M = 14.48 SE = 1.92). Nine out of the twelve CB participants improved between the training phase and testing phase of the experiment in terms of their Learning Rate of Detection (LRD: M = 9.45, SE = 2.63), and for Learning Rate of Avoidance (LRA) only two out of the twelve participants did not improve (M = 21.09, SE = 5.13). There was no significant difference between the scores for LRD (t(32) = 1.601, p = 0.060), and for LRA (t(32) = 1.44, p = 0.079) compared to SC. Of note, LRD and LRA were not correlated, meaning that the learning rate of obstacle detection is not linked to obstacle avoidance.

#### Principal component analysis of the brain volumes related to learning rate of detection (LRD)

Bivariate correlations identified 38 volumes of brain areas related to LRD. Principal components analysis revealed three different factors that explained 80% of the behavioral variance. The first factor explained 68% of the variance, the second one 6% and the third one 5%. These three components gathered the following brain regions: (1) **Medial Temporal Lobe Factor (MTL):** The first factor was composed of hippocampal and medial temporal lobe regions: Cornu Ammonis bilaterally, Entorhinal cortex on the right side, and the Subiculum bilaterally. (2) **Sensorimotor Factor (SM):** The second factor was composed of the following brain volumes: InferiorParietal (PFt) (supramarginal gyrus) on the right side, Brodmann area 3a (BA3a) of the human somatosensory cortex on the left side, and Brodmann area 4p (BA4p) on the left side in the primary motor cortex. (3) **Attention Factor:** The third factor included: The superior parietal lobule area 5 L (SPL 5 L) on the left side, Superior parietal lobule area (5Ci) bilaterally, and Brodmann area 4a (BA4a) on the left side.

The Cronbach’s alphas that are the three groups of variables composing the three factors appeared high (respectively: 0.949; 0.965; 0.905). This allowed for determining the composite variables from the independent variables in the three principal components.

**Figure 2:**
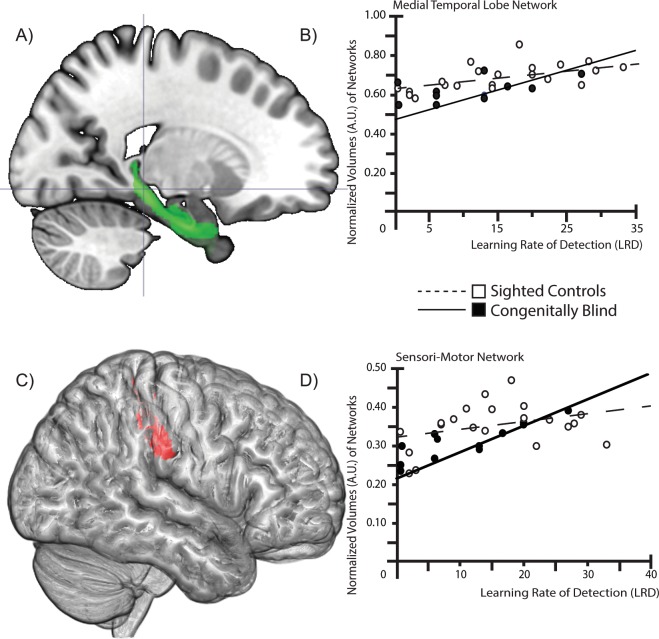
Obstacle Detection is Mediated by Different Networks for the Blind and Sighted. (**A**) Sagittal view showing hippocampal and entorhinal areas composing the Medial temporal Lobe factor. (**B**) Scatter plot showing the correlation between LRD and normalized volumes (in arbitrary units, A.U.). For SC, there was a significant correlation between the volume of this network and the LRD. (**C**) Sagittal view (left brain) showing areas composing the SensoriMotor factor. (**D**) Scatter plot showing the correlation between LRD and normalized brain volumes. For CB, there was a significant correlation between the volume of this network and the LRD.

In the case of SC individuals, the Medial Temporal Lobe factor explained 38% of the variance and was significantly correlated to LRD (beta = 0.62, t = 3.42, p = 0.003). The addition of the SM and the attention factors did not significantly increase the regression model (SM: ΔR2 = 0.02, ΔF = 0.47, p = 0.50; SM + Attention: ΔR2 = 0.00, ΔF = 0.00, p = 0.97). For CB subjects, MTL and SM factors explained 75% of the variance (compared to MTL only: ΔR2 = 0.38, ΔF = 12.33, p = 0.008). SM factor correlated significantly with LRD (beta = 0.66, t = 3.51, p = 0.008) but this was not the case for MTL (beta = 0.36, t = 1.93, p = 0.09) (Fig. 2). The inclusion of the attention factor in the model did not improve the explained variance (ΔR2 = 0.00, ΔF = 0.11, p = 0.75). Hierarchical regression modeling showed that the slopes were significantly different between CB and SC groups for the SM network (t = 2.38, p = 0.025) whereas not for MTL regions (t = 1.11, p = 0.28).

#### Principal component analysis of the brain volumes related to learning rate of avoidance (LRA)

Five brain volumes were correlated to LRA. The principal component analysis on these five variables showed one single factor - the Dorsal Stream - that explains 74% of the variance. The Dorsal Stream Factor was composed of mainly dorsal stream occipital and parietal areas: V3 (hOC3v) on the right side, V4 (hOC V4) on the right side, the Inferior Parietal Cortex (IPC PFm) on the left side, the superior parietal lobule area 7 M (SPL 7PC) on the right side, and 5 M (SPL 5 M) on the left side. All five variables were included in the factor (loadings are superior to 0.66). The Cronbach alpha for these variables was 0.90, which allowed averaging them and creating a composite variable.

**Figure 3:**
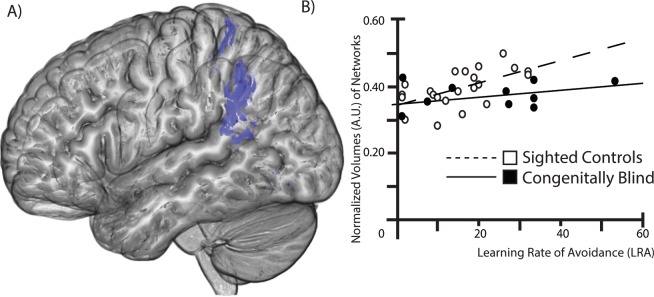
Obstacle Avoidance is Mediated by the Dorsal Stream Network: (**A**) Sagittal view (left brain) showing areas composing this factor. (**B**) Scatter plot showing the correlation between LRD and normalized volumes (in arbitrary units, A.U.). For both SC and CB, there was a significant correlation between the volume of this network and the LRA.

For the SC, 30% of the variance was explained by the dorsal stream factor and was significantly correlated to LRA (beta = 0.55, t = 2.85, p = 0.01). In the case of CB individuals, we found that 49% of the variance was explained by the dorsal stream network (beta = 0.70, t = 2.93, p = 0.02) (Fig. 3). Hierarchical regression modeling showed that the slopes were significantly different between CB and SC groups for the dorsal stream network (t = 2.51, p = 0.02).

Table 1 summarizes volumetric differences of the brain networks between SC and CB, and the correlations between the brain networks and performances. Of note, we did not find any significant correlation between the volumes of these brain networks and the baseline performances (obstacle avoidance or detection) neither for SC nor CB.

**Table 1:** Brain network characteristics and differences between sighted and congenitally blind groups.

Network	Task	Brain Regions	Volume Difference	Correlation with Learning Rates
Medial Temporal Lobe	Detection	Cornu Ammonis (LR)	P < 0.01	*LRD for SC* Beta = 0.62 t = 3.42 P = 0.003
		Enthorhinal Cortex (R)		
		Subiculum (LR)		
		Fascia Dentata (LR)		
Sensori-Motor	Detection	Inferior Parietal Cortex (Pft, R)	P = 0.02	LRD for CB* Beta = 0.66 t = 3.51 P = 0.008
		Area 3a (L)		
		Area 4p (L)		
Dorsal Stream	Avoidance	Superior Parietal Lobule (5 M, L)	P = 0.20	LRA for CB* LRA for SC* Beta = 0.55 Beta = 0.70 t = 2.85 t = 2.93 P = 0.01 P = 0.02
		Superior Parietal Lobule (7 pc, R)		
		Inferior Parietal Cortex (PFm, L)		
		hOC (V3, R)		
		hOC (V4, R)		

L: left, R: right, CB: congenital blind, SC: sighted control, *slopes are significantly different (P < 0.05).

## Discussion

In this study, we report that CB have a similar learning capabilities to SC for detection (LRD), and avoidance (LRA) of obstacles. In addition to the hippocampus and entorhinal cortex, LRD seems to involve a sensorimotor network in CB. As expected, for LRA and since there are no volumetric differences in the dorsal stream structures between CB and SC, this brain network is likely to be recruited in both groups. A possible limiting factor of our study is the relatively small sample size of CB participants. However, since CB individuals represent an exceptionally rare population, even more so when strict selection requirements are enforced, a sample of 12 participants can be considered as large and within the range of other classical brain morphometry studies in this population (for review see:^34^).

### Brain volumes and learning rate of detection correlations

Obstacle detection and avoidance in the context of normal vision is an *automatic* process that is still preserved in the absence of the visual cortex or visual awareness. This unconscious process is thought to require a subcortical visual input^35–37^. If then, the visual cortex is not necessary for obstacle avoidance, what cortical areas are then crucial for detection and avoidance of obstacles in CB? Obstacle avoidance relies on a complex and sophisticated avoidance system that is ‘sensitive and conservative’ in adjusting movement according to potential obstacles in the way^38^. This complex network of regions includes the hippocampus^39,]^^40^ and the visual dorsal stream^37^. In our experiment, obstacle detection learning was correlated with regions of the Medial Temporal Lobe for SC (hippocampus and entorhinal cortex). In CB participants, there is a large-scale anatomical reorganization of almost all brain regions^11,15,41^ and more specifically, the hippocampus that shows volumetric alterations^9–11^. We report here that, in addition to the hippocampus, CB recruit a sensorimotor network when learning the detection task. Our results fit remarkably well with recent literature on visually guided behavioral learning^42–45^, and spatial learning^46–48^. It is well known that brain volume can be associated with learning behavior^49,50^. A recent study also shows that hippocampal and entorhinal volumetric differences mediate spatial learning^46^, lending more support to the idea that the hippocampus, entorhinal cortex, and thalamus are important structures for navigation. In a recent study, memory guided attention task, hippocampal volume was related to the ability to implicitly learn contextual information^47^, and cognitive mapping style is mediated by the ratio of anterior-posterior hippocampal volume^48^. Moreover, our results are in line with the idea that the sensorimotor cortex is important for the plasticity of associative sensory learning^42^. A recent *f*MRI experiment also supports this view by showing that this same network is recruited during visually guided spatial behavior^43^. Furthermore, several recent studies have linked hippocampal volumetric differences in healthy^47^, and clinical populations with the ability to learn^49^. A recent study also demonstrated traces of learning in thalamo-cortical circuits^42^, and the impact of sensorimotor networks in guiding learning behavior^44^.

In the present study, we found that better learning *detection* performance was correlated with a larger hippocampus. Previous reports^51^ found respectively volumetric enhancement of the hippocampus head in CB and late blind participants^10^, while we had reported a volumetric reduction in the hippocampus tail of CB^9^. These differences have both been confirmed to co-exist in the hippocampus of CB by other reports^11,]^^15^. Moreover, there is impaired learning of object location (such as obstacles in a hallway) in human patients with hippocampal damage^52^. Spatial memory depends on synaptic plasticity of the hippocampus^53,]^^54^ and longer-lasting spatial memory is associated with a larger hippocampus^55^. For example, the posterior segment of the hippocampus is volumetrically enhanced in humans with extensive navigational training^39^. When CB participants were tested for route recognition in an fMRI scanner using a sensory substitution device, they recruited regions adjacent to the hippocampus and the parahippocampal place area^31^.

### Learning rate of avoidance and the dorsal stream network

The visual dorsal stream is important for controlling visually guided actions^56^ and selecting a path through a cluttered environment guided by vision^57^. For example, optic flow which is processed in area hMT+ and is an integral part of the visual dorsal stream provides information about the direction of self-motion and is very important for obstacle detection and avoidance^58^. Lesions to the dorsal stream^38,]^^59^, severely disrupts the ability to avoid obstacles, but lesions to the ventral stream do not^60^.

The posterior parietal cortex, which is also part of the dorsal visual stream, is involved in the processing of higher order spatial-cognitive information^7^. The parietal lobe is believed to register spatial information in interaction with the hippocampus^6,8^ and play a critical role in route decision making^61^. The posterior parietal cortex is also involved in visuo-spatial decision-making mediated by sensory motor integrations such as when pointing to a target^5^, and the visual guidance of movements in obstacle avoidance is a function of the occipito-parietal pathway^37^. We found correlations with parietal area 7 M. This area has been found to be sensitive to context dependent places in the macaque^62^, and activated in the human during execution and observation of an action^63^. The task of pointing to an obstacle and then avoiding it takes much planning indeed. After having pointed to the obstacle, one must keep in mind where the obstacle is located in space, and plan an appropriate response when arriving close to it^64^. In our study, there were no differences between SC and CB, in terms of their LRA performance, and although CB started out with a lower rate of avoidance in the training phase of the experiment, they performed as well as their SC counterparts in the test phase of the experiment, resulting in a higher LRA for CB compared to SC. Furthermore, although there was a volumetric reduction of almost all cortical and subcortical structures in CB, we find that the volume of the dorsal stream structures was preserved. We suggest that through mechanisms of brain plasticity, visual brain areas can be recruited to accomplish visuo-spatial tasks (just as they would do in the SC) using the visuo-tactile information provided by the sensory substitution device *in lieu* of vision.

## Conclusions

Our results indicate that there is a double dissociation between the brain networks involved in LRD and LRA for blind and sighted. Different networks are involved in the LRD behavior, namely for sighted individuals it is the medial temporal lobe network that is associated with this task, while for the blind it is certainly also sensorimotor areas that are associated with this behavior to achieve the same level of performance. Meanwhile, for both blind and sighted participants it is the dorsal stream network that is associated with their LRA behavior. It is interesting to note that the volume of this network is not affected by early blindness, as are almost all the other studied structures in CB.

## Methods

### Participants

Twelve right-handed CB participants (7 Males; average: 35; age range: 20–54 years old) with no history of light perception were compared to twenty-one normally SC participants (13 Males; average: 33; age range: 22–68 years old). All blind participants were recruited through the “Montreal Association for the Blind” and “Institut Nazareth et Louis-Braille”. Causes of blindness were retinopathy of prematurity in 4 cases; Glaucoma in two cases, congenital cataract, Leber’s amaurosis, detached retina, retinoblastoma, retinitis pigmentosa, and electrocution, each in one case, and a (medical) accident in two cases. The study was approved by the Ethics committee of the Université de Montréal in accordance with the Declaration of Helsinki. All subjects gave informed written consent prior testing.

### MR Image acquisition and volumetric analysis

Subjects were scanned in a 1.5 T Siemens MR Scanner (Siemens, Erlangen, Germany) in order to obtain fast spoiled grass gradient echo images using a standard head coil. Voxel-based morphometry was applied to 3D T1 MR images acquired in the axial plane, to assess purported correlations between the volume of brain regions known to be involved in navigation and performance. Sequence parameters were TI/TR/TE 450/106/42 ms, a flip angle of 20 degrees and a spatial resolution of 0.94 × 0.94 × 1 mm^3^, as previously reported in Ptito *et al*.,^15^. For Voxel Based Morphometry, spatial pre-processing of the brain scans was performed according to the optimized VBM protocol^65^ using SPM2 (Wellcome Department of Imaging Neuroscience, London, UK) and in-house software written in Matlab (MathWorks, Natick, MA, USA). For each subject, the averaged grey matter volumes were extracted within selected brain structures using some of the pre-defined probabilistic regions of interest included in the Anatomy Toolbox^66^: the medial temporal lobe^67,68^, hippocampus and surrounding areas^69^: Cornu Ammonis (CA), Fascia Dentata (FD), Hippocampal Amygdaloïd Transition Area (HATA) and Subiculum (SUB), BA 17 and 18, V3v, V4v, V5/MT+ ^70^, the superior parietal lobule (5ci, 5M, 7A, 7M, 7P and 7PC;^71^, hMT+ ^69^. The brain regions were selected because they are known to be involved in navigation from our own previous findings and other work (for review:^72^). Moreover, studies using the Shapiro-Wilk’s test of normality we find that the brain volumes were normally distributed for both CB (skewness = 0.501; p = 0.859) and SC (skewness = 0.637; p = 0.848).

### Visual-to-tactile sensory substitution system

**Figure 4:**
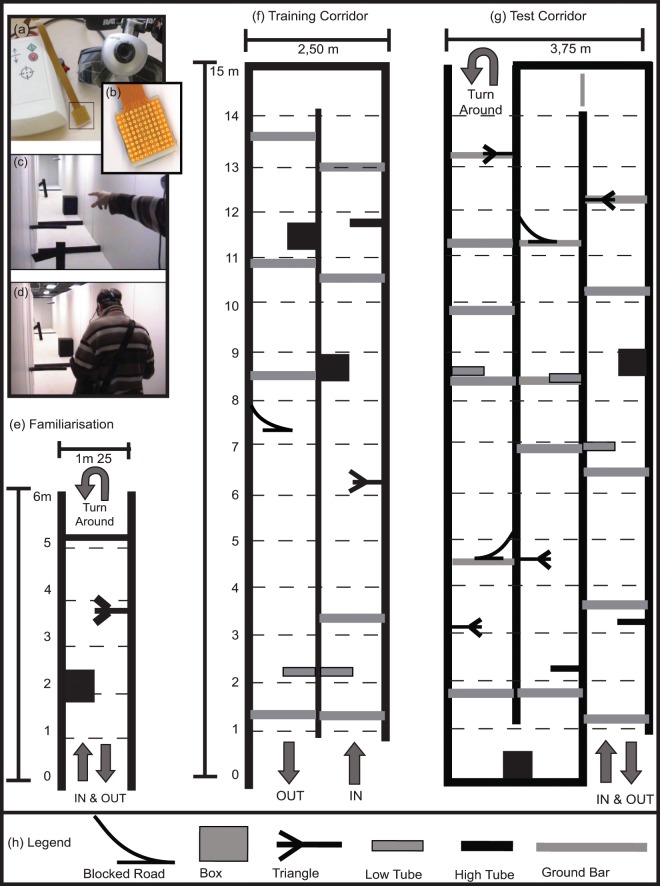
Apparatus and Schematic drawing of the experimental setup. (**a**) The tongue display unit (TDU) and Webcam mounted on a pair of safety goggles and fastened with an elastic band around the head. The image is translated into electro-tactile stimulation applied to the tongue using a 10 × 10 electrode array [boxed area in (**b**)]. The electrodes become active when a dark object enters the visual field of the camera. (**c,d**) An actual picture of a participant in the obstacle course pointing (**c**) and avoiding (**d**) obstacles. (**e**) Familiarization area with the juxtaposition of three different obstacles. (**f**) The training corridor includes two hallways of obstacles. (**g**) The testing corridor with three hallways. Hatched lines indicate 1 m distances. The exact location of the obstacles is represented with a different symbol for each obstacle type. (**h**) The different obstacle types.

We used a lingual visual-to-tactile sensory substitution system, the Tongue display Unit (TDU), that has been described in detail in earlier publications^15,27,33^. The TDU (Brainport, WICAB inc, Wisconsin, U.S.A.) consists of a webcam connected to a laptop computer and a tongue stimulator array (Fig. 4a,b). The tongue array consists of 100 small circular electrodes arranged in a 10 by 10 matrix with a diameter of 1 mm, and spaced 1 mm apart. The entire tongue array measures 2 × 2 cm (Fig. 4b). Participants wore the webcam mounted on a pair of light-tight safety goggles (Fig. 4a), in order to point and avoid the obstacles in the obstacle course (Fig. 4c,d). The camera sends images to the laptop which in turn converts them the visual information into electro-tactile pulses delivered to the tongue via an electrode array. Every time an object enters within the visual field, the visual image is translated into electro-tactile pulses that are transmitted to the tongue through the electrode array. The obstacles are thus ‘drawn’ with electrical current on the tongue in real time from the images provided by the webcam enabling then participants to point to and avoid obstacles.

### Experimental setup

A life-sized obstacle course (Fig. 4c–g), was used to assess the ability of congenitally blind and sighted control participants to detect and avoid obstacles with the TDU (Fig. 4a,b). Six different types of obstacles made of cardboard and styrofoam were used (Fig. 4h) (see Chebat *et al*.,^27^ for complete procedural details): (i) boxes (60 × 60 × 60 cm), (ii) triangles (60 × 45 × 2 cm), (iii) tubes (10 × 45 × 10 cm), (iv) branches (10 × 45 × 10 cm), (v) road blocks (150 × 10 × 10 cm) and (vi) ground bars (15 × 145 × 20 cm). They were made to resemble obstacles that can be realistically encountered on a sidewalk of any large city. The obstacles were positioned in each hallway according to Fig. 4. For the detection task, accurately pointing to an obstacle was scored as a correct response. For the avoidance task, negotiating a path without hitting or touching each obstacle was scored as a correct response after each obstacle was successfully passed. The obstacle course was composed of three main areas: the familiarization area (Fig. 4e), the training corridor (Fig. 4f) and the test corridor (Fig. 4g). In the familiarization phase, participants walked to the end of the corridor, turned around and came back three times, and each time obstacles were replaced in a different position. The number and positioning of the obstacles are outlined in Fig. 4. Participants were then placed at the entrance of the training corridor and walked down both corridors, and were placed at the entrance of the training corridor a total of four times. In the test phase of the experiment the participants walked through all three corridors, turned around and walked back to the entrance.

### Learning rate

We re-analyzed data accumulated in two previous experiments^15,27^, as well as novel and unpublished data in order to assess the learning rate of our participants in the detection and avoidance paradigm. Only those participants who had completed both behavioral testing and MRI scanning were included in the analysis. We compared their performances during the training phase with the test phase of the experiment. Learning rate was determined by subtracting the performance (percent correct responses) in the training phase of the experiment from the performance in the last phase of the experiment. The resulting scores were used to assess learning rate for detection (LRD) and the learning rate for avoidance (LRA).

### Statistical analyses

We used the SPSS statistical package (IBM SPSS Statistics 20 Software) for the analyses; i.e., t-tests, principal component analysis, and regression analysis. Differences between the learning rates (LRD and LRA) of SC and CB participants were evaluated using t-tests. We assessed the relation between the volumes of some specific brain areas and two dependent variables, LRD and LRA related to the ability of the participants to learn to detect and avoid obstacles in the obstacle course. This can be achieved with a regression analysis for each of the dependent variables. However, because of multicollinearity between brain region volumes (many were highly correlated), we performed two principal component analyses; one for the variables related to LRD and one for LRA. These analyses identify groups of independent and homogenous variables (forming brain networks in this case). Then, regression analyses can be performed since principal components (or factors) are independent from each other. These analyses evaluate the relationship between the volumes of brain areas and learning scores (LRD and LRA) for both SC and CB participants. We tested the strength of these relationships between the two groups in a hierarchical regression model (this approach is sometimes called Potthoff analysis^73,74^) including the principal components (or factors) obtained by PCA, an indicator for group (EB, SC) plus the products of the group with the factors. The test of homogeneity of slopes between groups is then obtained by a t-test for each factor^75^. Moreover, the most efficient model was evaluated for each subject group. To this aim, we tested different linear models including an increasing number of factors. Significant variations of R^2^ were evaluated using an F-test^75^ to select for the most efficient model.
